# A spliceosome-associated gene signature aids in predicting prognosis and tumor microenvironment of hepatocellular carcinoma

**DOI:** 10.18632/aging.204765

**Published:** 2023-06-10

**Authors:** Huaxiang Wang, Ruling Wang, Jian Fang

**Affiliations:** 1 Department of Hepatobiliary and Pancreatic Surgery, Taihe Hospital, Affiliated Hospital of Hubei University of Medicine, Shiyan 442000, Hubei, China; 2 Department of Hepatobiliary Medicine, The Third People’s Hospital of Fujian University of Traditional Chinese Medicine, Fuzhou 350108, Fujian, China

**Keywords:** hepatocellular carcinoma, spliceosome, signature, overall survival, tumor microenvironment

## Abstract

Splicing alterations have been shown to be key tumorigenesis drivers. In this study, we identified a novel spliceosome-related genes (SRGs) signature to predict the overall survival (OS) of patients with hepatocellular carcinoma (HCC). A total of 25 SRGs were identified from the GSE14520 dataset (training set). Univariate and least absolute shrinkage and selection operator (LASSO) regression analyses were utilized to construct the signature using genes with predictive significance. We then constructed a risk model using six SRGs (BUB3, IGF2BP3, RBM3, ILF3, ZC3H13, and CCT3). The reliability and predictive power of the gene signature were validated in two validation sets (TCGA and GSE76427 dataset). Patients in training and validation sets were divided into high and low-risk groups based on the gene signature. Patients in high-risk groups exhibited a poorer OS than in low-risk groups both in the training set and two validation sets. Next, risk score, BCLC staging, TNM staging, and multinodular were combined in a nomogram for OS prediction, and the decision curve analysis (DCA) curve exhibited the excellent prediction performance of the nomogram. The functional enrichment analyses demonstrated high-risk score patients were closely related to multiple oncology characteristics and invasive-related pathways, such as Cell cycle, DNA replication, and Spliceosome. Different compositions of the tumor microenvironment and immunocyte infiltration ratio might contribute to the prognostic difference between high and low-risk score groups. In conclusion, a spliceosome-related six-gene signature exhibited good performance for predicting the OS of patients with HCC, which may aid in clinical decision-making for individual treatment.

## INTRODUCTION

Hepatocellular carcinoma (HCC) is one of most prevalent cancer as well as the most lethal reason for cancer-associated mortalities on a global scale [1, 2]. Over the last few years, despite great advances on molecules and targeted immunotherapeutic drugs such as Sorafenib, Lenvatinib, Pembrolizumab, and Bevacizumab, the mortality rate of HCC remains unsatisfied high [3–6]. Inter- and intra-tumor heterogeneity were the great therapeutic challenges. In addition, the high recurrence rate further contributes to the high mortality and dismal survival probabilities, with a five-year recurrence of more than 70% and overall survival (OS) of less than 20% [7, 8]. Hence, identifying prognostic and therapeutic biomarkers is urgent for improving the clinical treatment and prognosis of HCC patients. STAT3, CTSA, SNRPD1, GP73, PIVKA-II, etc. were elucidated to be candidate prognostic biomarkers for HCC in multiple research [9–12]. Indeed, AFP, the golden standard molecular marker, was negative in approximately 30% of HCC [13]. It is now widely accepted that multiple genes’ signatures provide a better prognostic and diagnostic value than individual biomarkers. Applications of bioinformatics and machine learning methods have been developed and employed extensively to solve various complex challenges in recent years in disease diagnosis and treatment [14–17].

The splicing process of pre-messenger RNA-intron excision and exon ligation was the foundation of biodiversity and complexity in eukaryotic cells [18]. Spliceosome was responsible for the alternative splicing process [19, 20]. A large number of studies have implicated that abnormal splicing processes may lead directly to tumorigenesis and progression in a variety of tumors [21–23]. Quidville et al. suggested that after SNRPE and SNRPD1 knockdown by siRNA, two key components of the splicing process, the proliferative capacity and migratory ability of breast cancer cell lines were remarkably inhibited [24]. Wang et al. reported that SNRPD1 was remarkably highly expressed in HCC and promoted progression via the mTOR signaling pathway [25]. Hence, spliceosome-related genes (SRGs) may be the potentially effective prognostic biomarker for HCC patients. The effectiveness of multi-gene prognostic markers combined with the clinical characteristics was higher than single marker has been widely recognized [26, 27]. The prognostic significance of single SRGs in HCC has been extensively studied, but the multi-spliceosome gene signature has never been studied.

In this study, we recognized six SRGs to establish a signature from the GSE14520 dataset and validated its effectiveness in TCGA and GSE76427 datasets. Next, a predicting nomogram integrating with genes signature and some clinical predictors was constructed for of 1-, 3-, and 5-year OS quantitative prediction. Next, the decision curve analysis (DCA) was employed to take clinical decisions in practice. We then investigated the genetic alteration and immunohistochemistry staining of the six-SRGs signature in the cBioPortal and the Human Protein Atlas database. We also performed the gene set enrichment analysis to investigate the mechanism of these SRGs. Finally, we investigated correlations between gene signature and relative proportions of infiltrating immune cells.

## RESULTS

### Identification of differential expressed SRGs in the training set

**Figure 1:**
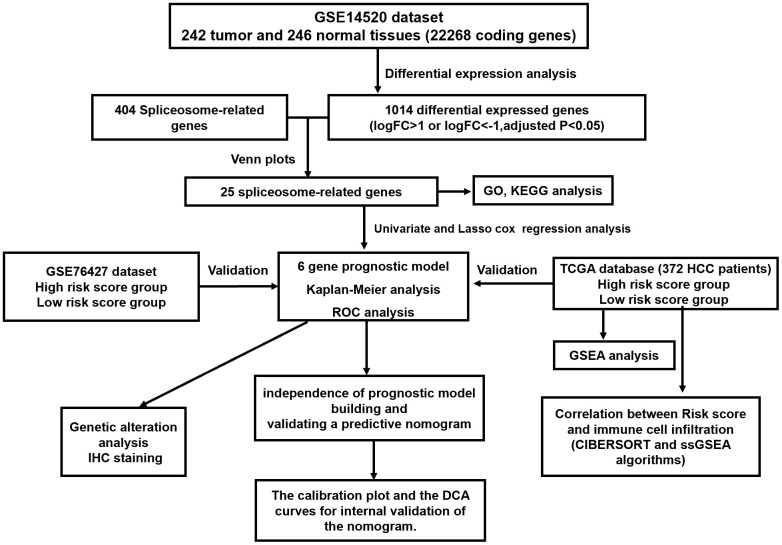
The flow chart exhibited the concise scheme of whole study.

The concise scheme of whole study was exhibited in a flow chart (Figure 1). 575 up-regulated and 539 down-regulated genes were recognized by comparison of HCC tissues with non-tumor tissue. Concurrently, we obtained 404 SRGs from the previously published article. Finally, the overlapping 25 SRGs between 1014 differentially expressed genes and 404 SRGs were identified as differentially expressed SRGs.

### Construction of the prognostic signature

**Figure 2:**
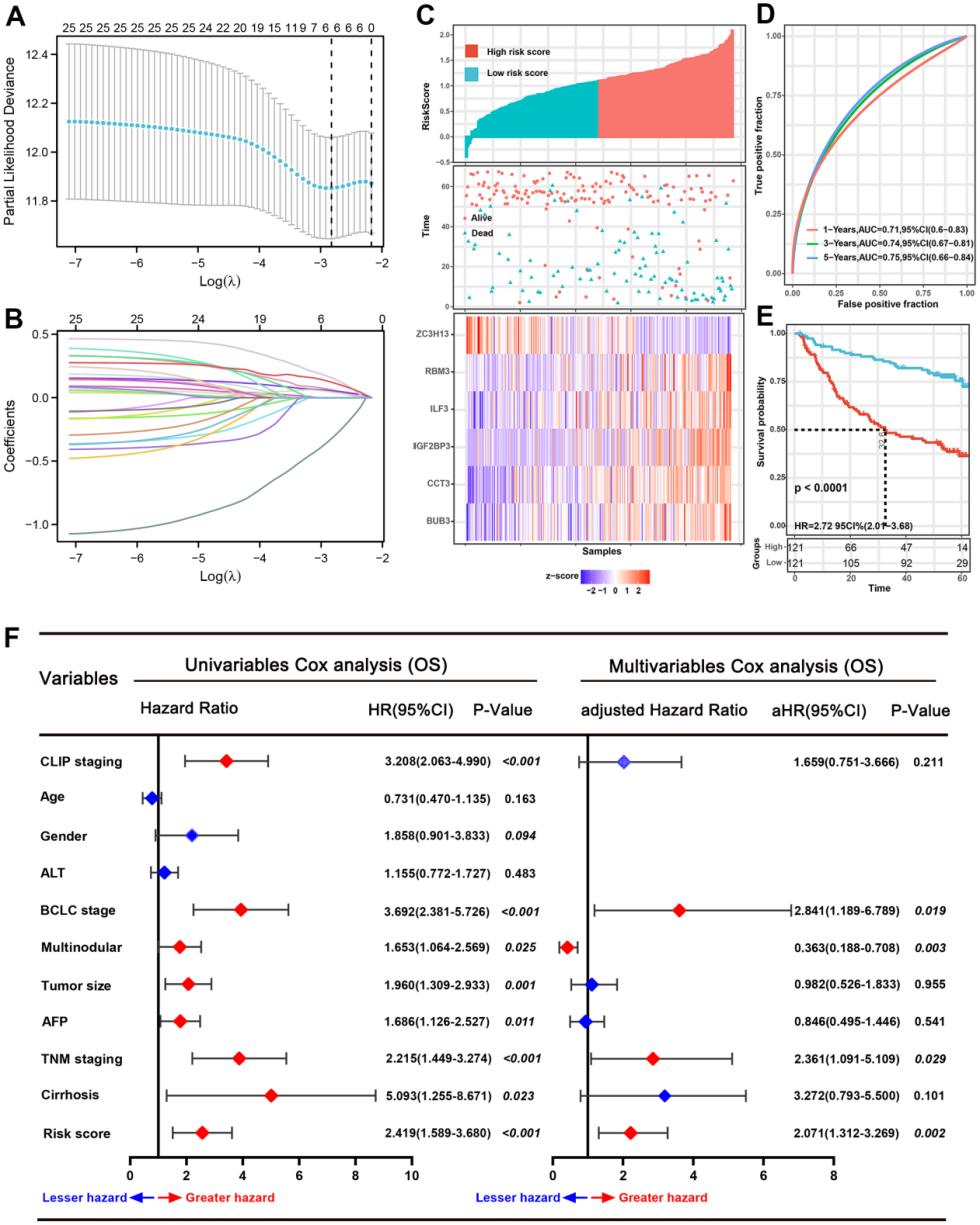
**Prognostic analysis of the six-spliceosome-related gene signature in the training set.** (**A**) six spliceosome-related genes were identified by the Least absolute shrinkage and selection operator (LASSO) regression model according to minimum criteria. (**B**) The coefficient of spliceosome-related genes was calculated by LASSO regression. (**C**) HCC patients were divided into high-risk and low-risk score groups based on the median risk score; High-risk score groups had lower survival rates than patients in low-risk score groups; Patients in high-risk score groups has lower ZC3H13 mRNA expression, whereas higher RBM3, ILF3, IGF2BP3, CCT3, and BUB3 mRNA levels. (**D**) 1-, 3-, and 5-year time-dependent receiver operating characteristic curve (ROC) of the spliceosome-related genes signature in the training cohort. (**E**) High-risk score patients have poorer overall survival probability than patients with low-risk scores in the training set. (**F**) Forrest plot of the univariate and multivariate Cox regression analyses for overall survival in the training set.

Univariate Cox regression and LASSO regression model analysis were applied to recognize the SRGs that impact prognosis. After the Lasso-Cox analysis, six genes were screened out to construct a signature (Figure 2A, 2B), and six genes were BUB3, IGF2BP3, RBM3, ILF3, ZC3H13, and CCT3. The risk score of each patient was counted according to an optimal λ value: risk score= 0.0618 * Exp BUB3 + 0.0538 * Exp IGF2BP3 + 0.0428 * Exp ILF3 + 0.1552 * Exp RBM3 + (-0.3199) * Exp ZC3H13 + 0.1208 * Exp CCT3.

### Validated the gene signature in the training set

According to the median risk score value, 242 HCC patients in the training set were divided into high-risk and low-risk score groups (Figure 2C). Next, the time-dependent ROC curve and the K-M survival analysis were applied to assess predictive performance of the genes signature for OS. The AUC for 1-, 3-, and 5-year OS prediction was 0.71, 0.74, and 0.75, respectively (Figure 2D). In addition, the Kaplan–Meier curve demonstrated that patients with high-risk score have poorer OS probabilities than patients with low-risk score (Figure 2E). Correlation analysis of risk scores with clinicopathological features found that the risk scores linked to TNM stage (P=0.029), AFP level (P<0.001), BCLC stage (P=0.029), and survival status (P<0.001) (Table 1). We then performed Cox proportional hazards regression analysis to recognize the independent risk factors of OS prediction. Univariate proportional hazards analyses suggested that CLIP staging, BCLC staging, multinodular, tumor size, serum AFP level, TNM staging, cirrhosis, and the high-risk score were risk factors for prognosis. Moreover, multivariate proportional hazards analyses suggested that BCLC staging (aHR (95%CI): 2.841(1.189-6.789); P=0.019), multinodular (aHR (95%CI): 0.363(0.188-0.708); P=0.003), TNM stage (aHR (95%CI): 2.361(1.091-5.109); P=0.029), and high-risk score (aHR (95%CI): 2.071(1.312-3.269); P=0.002) were independent risk factors for prognosis prediction (Figure 2F).

**Table 1:** Correlation between risk score and clinicopathological features of HCC patients for OS in the GSE14520 dataset.

**Characteristics**		**N**	**Risk score level**		***X* ^2^**	*P-Value
			**Low**	**High**		
Age	≥55	83	42	41	0.018	0.892
	<55	159	79	80		
Gender	Male	211	103	108	0.925	0.336
	Female	31	18	13		
Main tumor size	≥5cm	88	38	50	2.571	0.109
	<5cm	154	83	71		
TNM stage	I-II	174	95	79	4.746	0.029
	III-IV	51	19	32		
Serum AFP level	≥300ng/ml	110	41	69	13.158	<0.001
	<300ng/ml	128	79	51		
ALT	≥50U/L	100	52	48	0.273	0.602
	<50U/L	142	69	73		
Multinodular	Yes	52	26	26	<0.001	1.000
	No	190	95	95		
Cirrhosis	Yes	223	108	115	2.799	0.094
	No	19	13	6		
CLIP score	≥2	48	19	29	2.999	0.083
	<2	177	95	82		
BCLC stage	B-C	53	20	33	4.790	0.029
	0-A	173	95	78		
Survival status	Dead	96	34	62	13.537	<0.001
	Alive	146	87	59		

OS, overall survival; TNM, tumor, node, metastasis; AFP, alpha fetoprotein; ALT, alanine aminotransferase; BCLC, Barcelona clinic liver cancer; *P-Value <0.05 were considered statistically significant.

### Verified the gene signature in two external datasets

**Figure 3:**
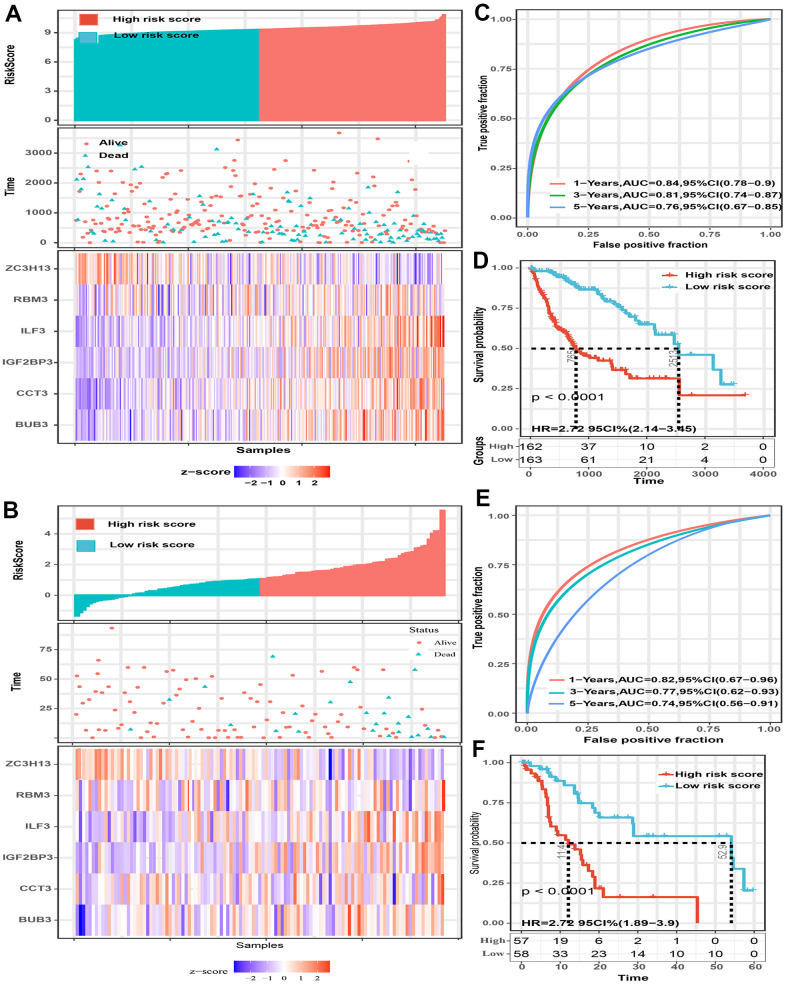
**The predictive value of the six-spliceosome-related gene signature was validated in two validation sets (TCGA and GSE76427 cohorts).** (**A**, **B**) HCC patients were divided into high-risk and low-risk score groups based on the median risk score in the TCGA cohort (**A**) and GSE76427 cohort (**B**); HCC patients were divided into high-risk and low-risk score groups based on the median risk score; High-risk score groups had lower survival rates than patients in low-risk score groups; Patients in high-risk score groups have lower ZC3H13 mRNA expression, whereas higher RBM3, ILF3, IGF2BP3, CCT3, and BUB3 mRNA levels. (**C**) 1-, 3-, and 5-year time-dependent receiver operating characteristic curve of the spliceosome-related genes signature in the TCGA cohort. (**D**) High-risk score patients have poorer overall survival probability in the TCGA cohort. (**E**) 1-, 3-, and 5-year time-dependent receiver operating characteristic curve of the spliceosome-related genes signature in the GSE76427 cohort. (**F**) High-risk score patients have poorer overall survival probability in GSE76427 cohort.

**Figure 4:**
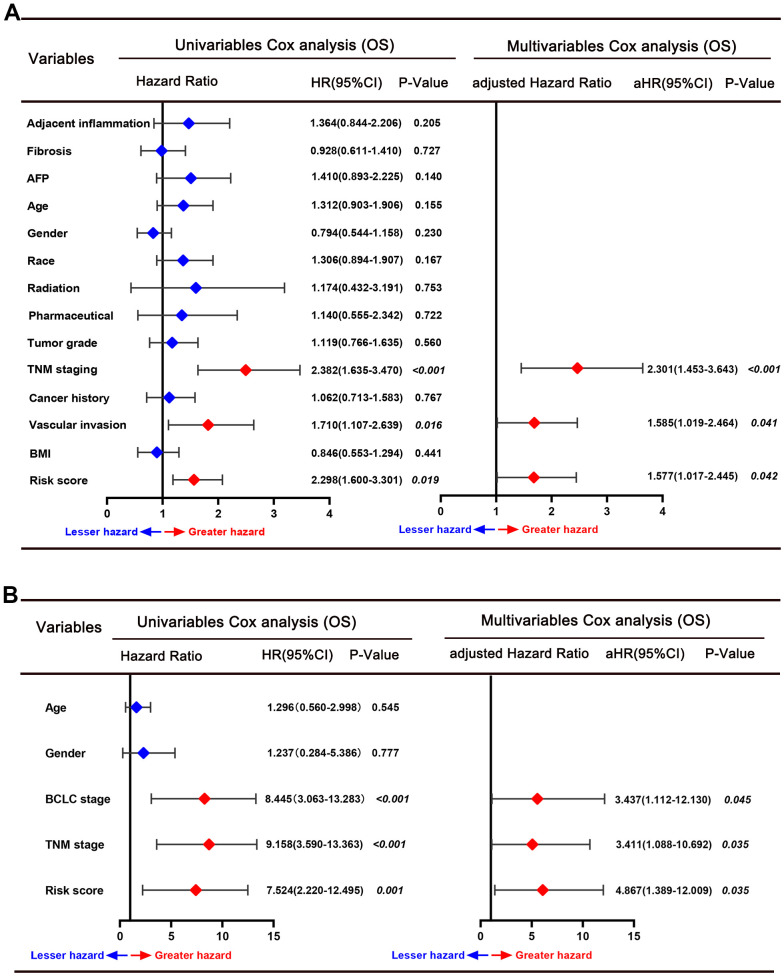
Forrest plot of the univariate and multivariate Cox regression analyses for overall survival in the TCGA cohort (**A**) and GSE76427 cohort (**B**).

The predictive power of this six-gene signature next was validated in two external datasets (TCGA and GSE76427 dataset). Patients in two external validation sets were divided into high and low-risk score subgroups according to the median value of risk score, respectively (Figure 3A, 3B). The AUC for 1-, 3-, and 5-year OS prediction in TCGA set was 0.84, 0.81, and 0.76, respectively (Figure 3C). Furthermore, the survival curve demonstrated that patients with high-risk score have poorer OS probabilities than patients with low-risk score (Figure 3D). Our analysis indicated a remarkable correlation of high-risk score with gender (P=0.042), tumor grade (P<0.001), TNM staging (P=0.008), AFP (P<0.001), and cancer history (P=0.002) (Table 2). The AUC for 1-, 3-, and 5-year OS prediction in GSE76427 validation set was 0.82, 0.77, and 0.74, respectively (Figure 3E). As same to the training set, the K-M curve indicated that patients with high-risk score have a significantly shorter OS (Figure 3F). A significant association between high-risk scores with worse clinicopathological outcomes (BCLC staging, TNM staging, and survival status) was still validated in this HCC cohort (Table 3). Cox proportional hazards regression analysis was also applied to assess the predictive ability of the signature in two validation sets. Results demonstrated that this predictive signature was determined as an independent predictor for OS via multivariate hazards analysis (TCGA cohorts: P=0.042; GSE76427 cohorts: P=0.035) (Figure 4A, 4B).

**Table 2:** Correlation between risk score and clinicopathological features of HCC patients for OS in the TCGA HCC cohort.

**Characteristics**		**N**	**Risk score level**		***X* ^2^**	*P-Value
			**Low**	**High**		
Age	≥60	119	60	59	0.025	0.875
	<60	206	102	104		
Gender	Male	216	99	117	4.148	0.042
	Female	109	63	46		
Race	White	157	101	56	25.490	<0.001
	Other	168	61	107		
Tumor grade	G1-G2	204	117	87	12.351	<0.001
	G3-G4	121	45	76		
Radiation	Yes	9	5	4	0.121	0.728
	No	316	157	159		
Pharmaceutical	Yes	17	10	7	0.578	0.447
	No	308	152	156		
TNM staging	I-II	238	129	108	6.930	0.008
	III-IV	87	33	54		
Adjacent inflammation	NO	111	65	46	1.533	0.216
	Yes	119	60	59		
Serum AFP level	≥300ng/ml	68	20	48	16.494	<0.001
	<300ng/ml	224	118	86		
Fibrosis	Yes	166	75	91	6.747	0.009
	No	106	65	41		
Cancer history	No	186	81	105	9.537	0.002
	Yes	97	61	36		
Vascular invasion	NO	181	95	86	0.153	0.696
	Yes	94	47	47		
Survival status	Dead	113	51	62	1.539	0.215
	Alive	212	111	101		

OS, overall survival; TNM, tumor, node, metastasis; AFP, alpha fetoprotein; *P-Value <0.05 were considered statistically significant.

**Table 3:** Correlation between risk score and clinicopathological features of HCC patients for OS in the GSE76427 dataset.

**Characteristics**		**N**	**Risk score level**		***X* ^2^**	*P-Value
			**Low**	**High**		
Age	≥60	67	38	29	3.284	0.070
	<60	48	19	29		
Gender	Male	93	48	45	0.815	0.367
	Female	22	9	13		
BCLC staging	0-A	80	45	35	4.699	0.030
	B-C	35	12	23		
TNM staging	I-II	82	45	37	4.372	0.037
	III- IV	33	11	22		
Survival status	Dead	24	4	20	13.131	<0.001
	Alive	91	53	38		

OS, overall survival; TNM, tumor, node, metastasis; BCLC, Barcelona clinic liver cancer; *P-Value <0.05 were considered statistically significant.

### Establishment and detection of a predictive nomogram in training set

**Figure 5:**
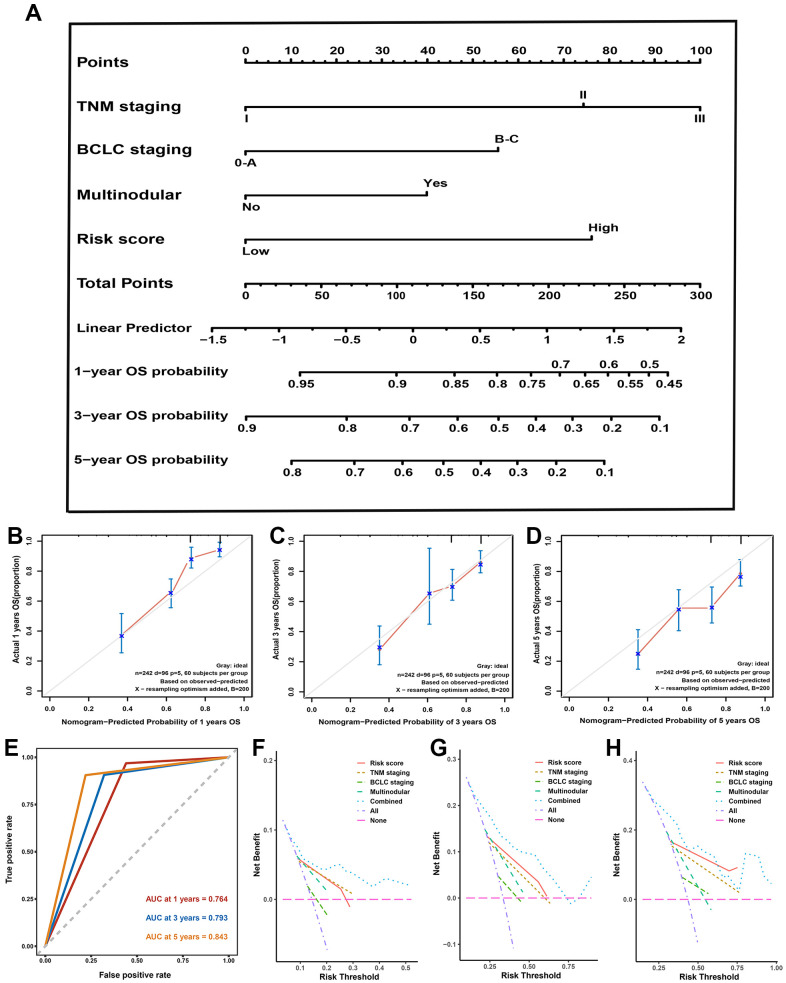
**Nomogram, calibration plot, decision curve analysis in training set.** (**A**) Predictive nomogram for prediction of 1-, 3-, and 5-year overall survival rates. (**B**–**D**) The calibration curve to detect the predictive performance of nomogram in 1- (**B**), 3- (**B**), and 5-year (**C**) overall survival rates. (**E**) The time-dependent Receiver operating characteristic curve for 1-, 3-, and 5-year to validate the predictive performance of nomogram. (**F**–**H**) The decision curve analysis exhibited the highest net benefit of the nomogram for 1- (**F**), 3- (**G**), and 5-year (**H**) overall survival rates.

All independent predictors recognized by hazards regression analyses from the GSE14520 dataset were integrated for predictive nomogram construction and prediction of 1-, 3-, and 5-year OS probabilities. Our results suggest that multinodular, TNM staging, BCLC staging, and risk score were independent predict factors, so the nomogram included these features (Figure 5A). Calibration curves and ROC curves were employed to detect the predictive ability of the nomograms. Compared with ideal model, the nomogram exhibited an excellent prediction performance for OS probabilities at different years (Figure 5B, 5D). AUC for 1-, 3-, and 5-year OS probability predictions for the predictive model were 0.764, 0.793, and 0.863, respectively (Figure 5E). Moreover, the DCA curve revealed that the predictive model showed a better net benefit for 1-, 3-, and 5-year OS rates (Figure 5F–5H). Our results demonstrated that this hybrid nomogram encompassing the predictive signature with clinicopathological features has excellent stability and accuracy and can play a role in HCC patient management.

### Genetic alteration of gene signature correlated to worse OS

**Figure 6:**
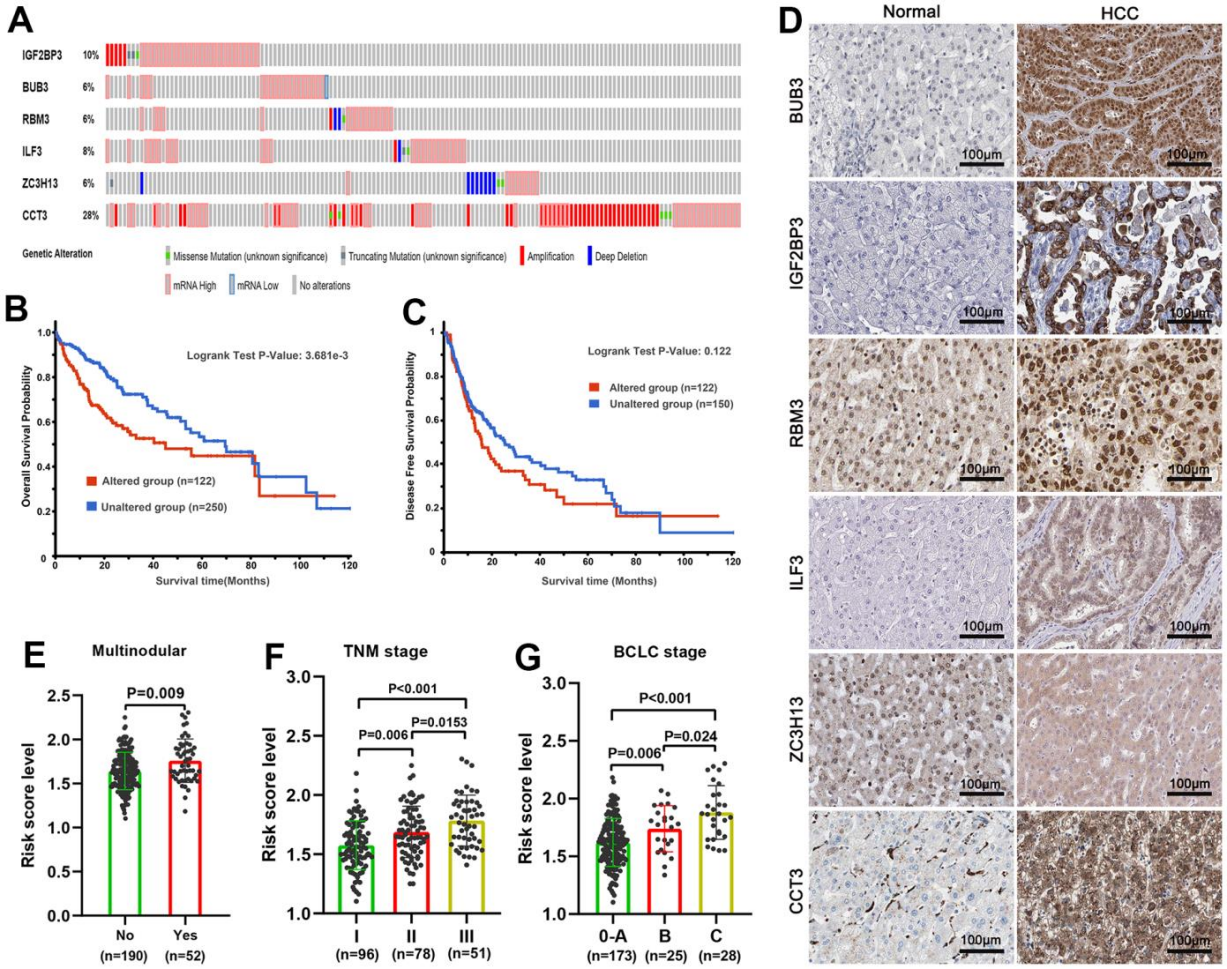
**Genetic alteration and protein expression analysis of six spliceosome-related genes signature in HCC.** (**A**) The summary of genetic alterations of each spliceosome-related gene in the cBioPortal database. (**B**) Patients with genetic alteration have poorer overall survival probability. (**C**) There was no statistical difference in disease-free survival probability between the patients with genetic alteration and without genetic alteration. (**D**) The representative protein expression and localization of the BUB3, IGF2BP3, RBM3, ILF3, ZC3H13, and CCT3 in HCC and adjacent normal liver tissues. (**E**–**G**) The risk score was incrementally increased with increasing tumor nodular (**E**), TNM staging (**F**), and BCLC staging (**G**) in the training set.

We queried the gene-altered information of the SRGs signature using TCGA, PanCancer Atlas dataset in cBioPortal. Among 372 HCC patients detected, 122 patients (32.8%) exhibited the genetic alterations in this signature (Figure 6A). Moreover, compared with patients without genetic alteration, genetic alteration patients had worse rate in OS, and the disease-free survival rate has not statistically significant (Figure 6B, 6C). We then queried the protein localization and quantification of the six SRGs in HCC and their paracancer tissues employing the Human Protein Atlas database. Results demonstrated that BUB3, IGF2BP3, RBM3, ILF3, and CCT3 proteins were remarkably increased in HCC tissues, while the ZC3H13 protein was decreased (Figure 6D). We then analyzed the risk score value in different stage tumor tissues in the training set and noticed an increasing tendency with tumor nodular, TNM staging and BCLC staging gradually increasing (Figure 6E–6G).

### Enrichment analysis of spliceosome-related genes via GO and KEGG

**Figure 7:**
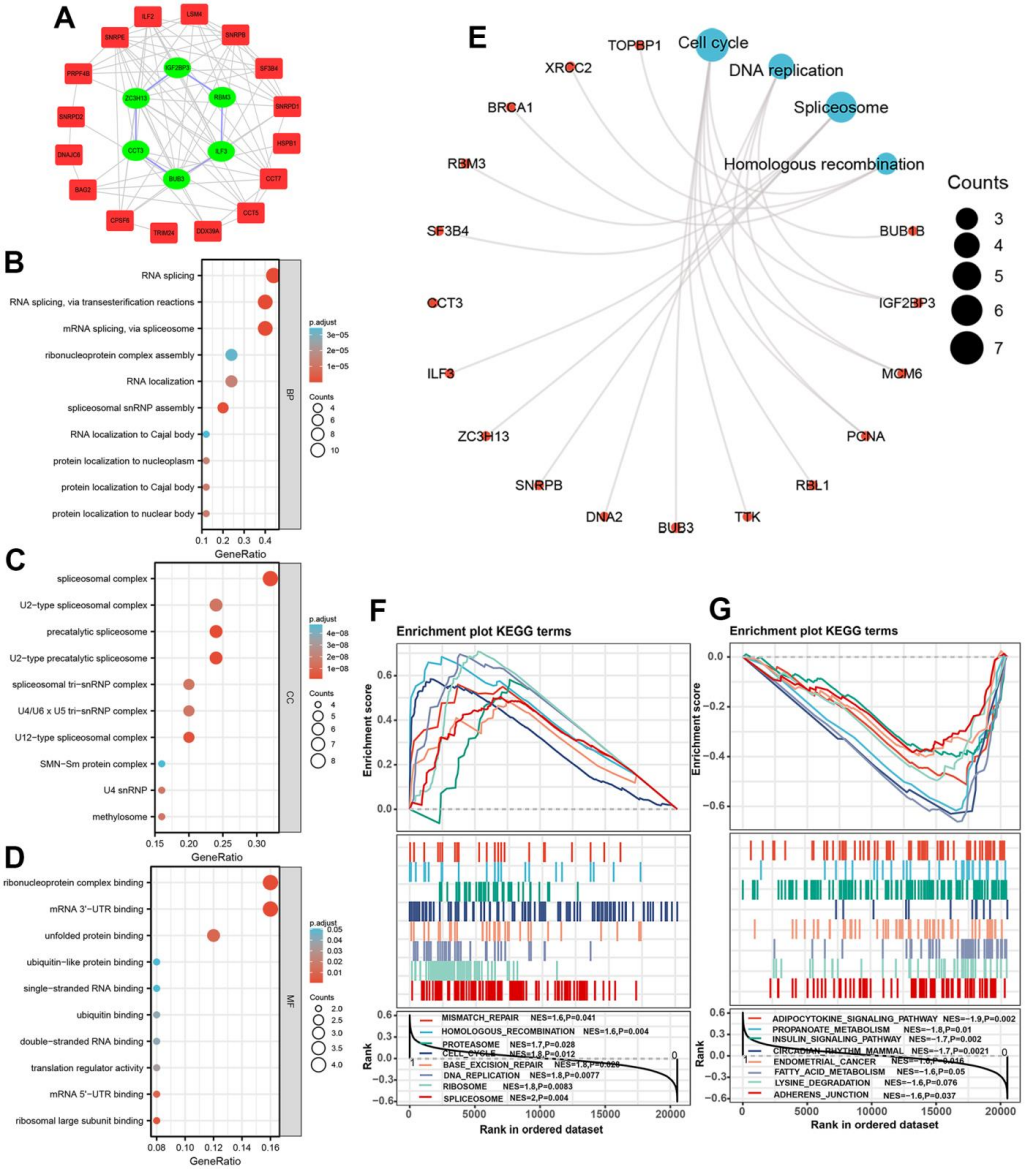
**Protein-protein interaction network and functional enrichment analysis of spliceosome-related genes.** (**A**) The protein-protein interaction network between 25 spliceosome-related genes. (**B**–**E**) The 25 spliceosome-related genes were mainly enriched in the biological process (**B**), cellular component (**C**), molecular function (**D**), and KEGG pathway (**E**). (**F**, **G**) GSEA identified the signaling pathways in which genes expressed in the high-risk (**F**) and low-risk score patients (**G**) enriched.

A PPI network reflecting the interaction between these SRGs was built and displayed by employing the Cytoscape version 3.7.1 (Figure 7A). We then analyzed GO and KEGG pathway enrichment analysis on 25 SRGs to explore the gene biological functions and related signal transduction pathways. For biological process terms, 25 SRGs were mainly enriched in the RNA splicing process, such as RNA splicing via transesterification reactions (Figure 7B). For cellular component terms, the 25 SRGs were found enriched in precatalytic spliceosome and spliceosomal tri-snRNP complex (Figure 7C). For molecular function terms, the ARGs were mostly enriched in ribonucleoprotein complex binding, mRNA 3’-UTR binding, and unfolded protein binding (Figure 7D). Further KEGG analyses suggested that these SRGs were mostly involved in Spliceosome signaling, Cell cycle, and DNA replication (Figure 7E).

### GSEA

GSEA analysis was implemented to explore intensively the significant signaling in which genes enriched in patients in different risk score subgroups. Results indicated that patients in high-risk score subgroup were associated with Spliceosome signaling (NES=2, P=0.004), DNA replication signaling (NES=1.8, P=0.008), Base excision repair signaling, and Cell cycle signaling (NES=1.8, P=0.012) (Figure 7F), whereas patients in the low-risk score subgroup were linked to Adherence junction signaling (NES=-1.6, P=0.037), Lysine degradation signaling (NES=-1.6, P=0.037), Adipocytokine signaling (NES=-1.9, P=0.002), Propanoate metabolism signaling (NES=-1.8, P=0.01), Insulin signaling (NES=-1.7, P=0.002), and Endometrial cancer signaling (NES=-1.6. P=0.016) (Figure 7G).

### Immune infiltration analysis in two subgroups

**Figure 8:**
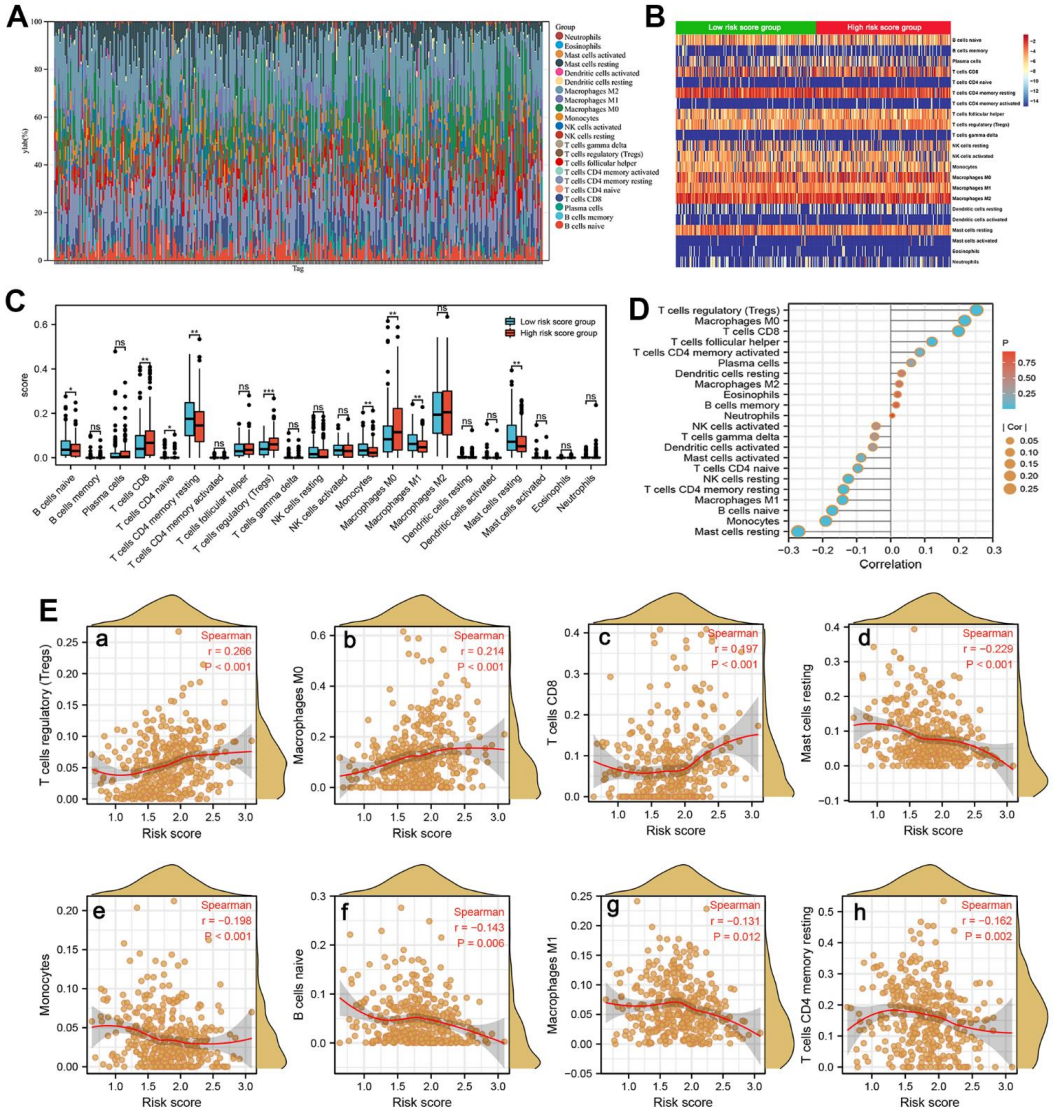
**Immune infiltration analysis.** (**A**) The estimated fractions of 22 immune cells in each HCC sample using the CIBERSORT algorithm and visualized in a bar plot. (**B**) The heat map exhibited the differences in the fraction of 22 immune cells between the high-risk score and low-risk score groups. (**C**) The comparison of estimated fractions of 22 immune cells between the high-risk and low-risk score groups. (**D**) The correlation between the risk score and 22 tumor-infiltrating immune cells in HCC using the ssGSEA algorithm. (**E**) The correlation of risk score with immune infiltration level of T cells regulatory (**a**), Macrophages M0 (**b**), T cells CD8 (**c**), Mast cell resting (**d**), Monocytes (**e**), B cells naive (**f**), Macrophages M1 (**g**), and T cells CD4 memory resting (**h**).

A growing body of evidence confirmed that the abundance of immunocyte infiltration and composition play a non-negligible role in tumorigenesis and cancer progression [28]. We counted the assessed fractions of 22 immunocytes of all HCC tissue employing the CIBERSORT method and visualized it in a bar plot. The assessed fraction of 22 immunocytes in each HCC sample added up to 1, with each color representing one type of immunocyte (Figure 8A). The differences in fraction of 22 immunocytes between the high and low-risk score subgroups were investigated and visualized in a heat map (Figure 8B). Moreover, our results indicated that patients in high-risk subgroup predicted a larger proportion of T cells CD8, T cells CD4 naive, T cells regulatory, and Macrophages M0, while a lower proportion of B cells naive, Macrophages M1 etc. (Figure 8C). Next, ssGSEA algorithm was used to investigate correlation between the risk score and 22 tumor-infiltrating immunocytes in HCC (Figure 8D). As shown in Figure 8E, the risk score positively correlated to T cells regulatory (r=0.266, P<0.001) etc. while was negatively associated to Mast cell resting, B cells naive, Macrophages M1, and T cells CD4 memory resting (Figure 8E), suggesting that these SRGs signature may be a regulator of immunocytes infiltration level in HCC.

## DISCUSSION

HCC is a common cancer type in the digestive system with poor prognosis [28]. The morbidity of HCC gradually decreased with the incidence reduction of hepatitis B and C in recent decades, but the mortality is still high, especially in sub-Saharan and other low-income countries [29, 30]. A growing number of published studies combined public databases with integrative bioinformatics analysis investigated the alterations of various types of molecules such as mRNA, miRNA, and lncRNAs to elucidate the potential mechanism and valuable signaling, identifying the biomarkers with diagnostic and prognostic values [26, 31, 32]. Recently, dysregulation of pre-mRNA splicing into mRNA increasing protein diversity has attracted extensive attention [20, 33]. The increasing number of evidences demonstrated that the alteration of SRGs can induce the abnormal of splicing process, resulting in the tumorigenesis and proliferation of HCC [34]. Several research have verified that the dysregulation by aberrant expression of many SRGs such as MTR4, SF3B1, CCT7, AND NUDT21 was tightly linked to the worse prognosis of HCC [35–38]. However, existing research mainly focused on the individual spliceosome-related genes in the progression of HCC, and systematic studies of multiple genes signature were lacking.

In our study, according to the relative transcription level in the training set, a signature based on 6-SRGs (BUB3, IGF2BP3, RBM3, ILF3, ZC3H13, and CCT3) was built for prognosis prediction of HCC. It was encouraging to note that one internal validation and two external validations both showed that patients with higher risk scores have a poorer survival probability than patients with lower risk scores. In addition, ROC curves for different years also display the great predictive capacity of the signature in training and validation sets. Moreover, our correlation results suggested that risk score of patients correlated to poor clinicopathological features, such as TNM staging, BCLC staging, multinodular, and survival status. Furthermore, multivariate Cox proportional hazards regression analysis in one internal validation and two external validation sets elucidated that high-risk score serves as an independent risk factor for HCC OS rate prediction. Compare with existing signatures, this SRGs signature display a larger AUC value for predicting 1-, 3-, and 5-year OS probabilities [39, 40].

We next establish a predictive nomogram which combined SRGs signature and other clinicopathological parameters to quantitatively predict OS rate. The predictive performance was validated by the calibration curve and DCA curve. In this research, the gene-altered data of the six-SRGs were queried and exhibited that with gene-altered patients have poorer OS probability. Therefore, it is reasonable speculated that the abnormal expression of these genes was driven by genetic alteration. Herein, the functional analysis by KEGG and GSEA elucidated that these SRGs were remarkably linked in the spliceosome-associated signaling and other tumor proliferation-associated pathways, such as DNA replication, base excision repair, and cell cycle, which plays an indispensable role in the liver tumorigenesis and development [41–43]. Our GSEA analysis showed several significantly enriched cancer-related signaling pathways since the number of Spliceosome-related genes is small and a larger number of genes is needed in future studies.

The subsequent immune infiltration analysis by CIBERSORT and ssGSEA algorithm. CIBERSORT outperformed other algorithms in respect of noise control, unknown mixture components, and tightly associated with cell type. Previous studies suggested that xCell algorithm can be used for calculation of fractions for immune cells, tumor cells and stromal cells and it superior to other extensive computer algorithms. [44, 45]. Herein, we just analyzed the infiltration abundance of immunocytes in tumor tissues with different risk scores, without involving stromal cells and other cells, so we did not employ the xCell algorithm. Our analyses indicated that patients in high-risk subgroup predicted a larger proportion of T cells CD8, T cells CD4 naive, T cells regulatory, and Macrophages M0, while a lower proportion of B cells naive, Macrophages M1 etc. Sun and colleagues showed that CD8 + T cells decreased innate-like low cytotoxic state and promoted recurrence by overexpressing KLRB1 (CD161) [46]. In addition, Regulatory naive CD4+ T-cells impair cancer immunosurveillance by creating an immunosuppressive environment, thereby promoting tumor progression [47, 48]. The decreased densities of tumor-infiltrating naive B cells in HCC imply a poor survival rate and was an independent prognosticator [49]. Therefore, the disordered infiltration of immune cells in HCC causes the dysfunctional immune response and may be a primary cause of the poor prognosis. Therapies designed to target immune cells may be new treatment avenues in HCC.

Our predictive signature was based on six spliceosome-associated genes. Among these genes, BUB3, IGF2BP3, RBM3, ILF3, and CCT3 were risk factors, while ZC3H13 played a protective role. BUB3 regulated mitosis of eukaryotic cells and has been closely related to tumorigenesis of a variety of cancers. A signature based on 5 cell cycle genes suggested that BUB3 can be a prognostic biomarker in HCC [50]. The role of IGF2BP3 in HCC has been extensively studied. Li Zhe et al. demonstrated that IGF2BP3 promotes aggressive and invasive ability of HCC by stabilizing the transcript of LIN01138 [51]. In addition, the reciprocal regulation between IGF2BP3 and HBV-pregenomic RNA drives the progression of HBV-Related HCC by increasing the stemness [52]. RBM3, a well-proven oncoprotein in a variety of cancers, promotes the proliferation of HCC cells by increasing YAP1 expression [53]. In addition, ILF3 and CCT3 have been confirmed to play a role of protumorigenic in HCC [54, 55]. ZC3H13 was a tumor suppressor gene in a variety of types of cancer. Knockdown of ZC3H13 significantly promotes aggressive and invasive ability of HCC cells, while upregulation inhibited the cell’s invasion and proliferation by regulating JAK-STAT signaling pathway [56]. Taken as a whole, our results are consistent with as mentioned in the above studies, suggesting that our results are reliable. However, there are some limitations in the presented analyses. First, the 6-SRGs signature and risk model was established based on one internal validating set and two external validating sets, and more validation sets across sources were needed. Second, the presented results were obtained by bioinformatic and public databases analysis and have not been verified by performing deeper Vivo and Vitro assays. Therefore, a large of clinical practice was required, which will be supported by *in vitro* cell experiments.

## CONCLUSIONS

In conclusion, this study established a predictive six-spliceosome-associated genes model and constructed a combined nomogram to quantitatively evaluate the OS rates of HCC. Our predictive model exhibited excellent prediction performance to guide clinical decisions and to select the optimal treatment plan for patients. In addition, the SRGs signature may be linked in infiltration of the immunocytes, thereby affecting the survival rates of HCC patients. Therapies designed to target immune cells may be new treatment avenues in HCC. However, a large of clinical practice was required, which will be supported by *in vitro* cell experiments.

## MATERIALS AND METHODS

### Gene expression acquisition and compilation

Transcriptomic and clinical characteristics data of patients were acquired from GSE14520, GSE76427, and the TCGA datasets. The list of SRGs was acquired from the previously published article and searched in PubMed [57]. The selection criteria of the datasets were following: (1) HCC was confirmed by pathology review for all patients; (2) the total number of tumor and adjacent normal liver samples are more than 100; (3) the clinicopathological and survival data were sufficiently complete; (4) patients had greater than 30 days follow-up. Based on the criteria, the GSE14520 dataset (training set, containing 242 HCC and 246 normal liver samples) was selected to construct the predictive signature. The TCGA (containing 325 HCC samples) and GSE76427 (containing 115 HCC samples) datasets were employed as the validation set. All transcriptomic and clinical characteristics data of patients were publicly available in the public database; thus, no additional ethical review in this research was required.

### Analysis of the differentially expressed SRGs

R software with the “limma” package was employed for the comparison of the transcription profiling mRNA data between HCC and normal liver samples in the training set (GSE14520). Differentially expressed genes with a |log2FC| greater than 1.2 and corrected P less than 0.05 were selected to subsequent analyses. Overlapping differentially expressed genes identified by the “limma” from the training set and SRGs were then extracted as differentially expressed SRGs.

### Built and verified the SRGs signature

Univariate Harzard regression and LASSO regression model analyses were applied to recognize the SRGs that impact prognosis and then built a gene signature for OS prediction. We next calculated each patient’s risk score according to the following formula: Risk score= (coef _gene 1_ * Exp _gene 1_) + (coef _gene 2_ * Exp _gene 2_) + (coef _gene 3_ * Exp _gene 3_) + … + (coef _gene n_ * Exp _gene n_) (coef can be obtained from the LASSO model). Next, we classified the patients into low- and high-risk score groups according to the median value of risk score. We compared the clinicopathologic characteristics between two risk score subgroups. In addition, the time-dependent ROC and K-M survival curve, as well as the Multivariate Harzard regression model was utilized to evaluate the predicting value of the SRGs signature. Finally, the reliability of this SRGs signature was verified using TCGA and GSE76427 dataset, respectively.

### Establishment and verification of the predictive nomogram

All independent risk factors of worse prognosis recognized by the Multivariate Hazard regression model in GSE14520 were integrated into a nomogram for exact prediction of 1-, 3-, and 5-year OS probabilities. Subsequently, the predictive ability of the nomogram at varying year points was evaluated by plotting the calibration curve and time-dependent ROC curve with the help of the “timeROC” R package in OS. We utilized the “ggDCA” R package to plot DCA curves and determine the optimal decision with maximum clinical net benefit.

### Gene-altered analyses and immunohistochemical staining of SRGs

Genetic alteration and mutations often cause gene expression changes that result in tumorigenesis and cancer progression [58]. We quired the copy number changes and mutations of SRGs signature by employing PanCancer Atlas set in the cBioPortal database [59, 60]. Subsequently, we determined the OS and disease-free survival probabilities by comparing groups with genetic alterations and those without. We also studied the SRGs protein quantification dissimilarity in HCC and paracancer liver tissue through immunohistochemistry analysis in the Human Protein Atlas database [61].

### GO, KEGG, and GSEA enrichment analyses

To elucidate the potential role and molecular mechanism, the “clusterProfiler” package was employed for GO and KEGG pathway enrichment analyses on 25 SRGs. The significance level was set at p < 0.05 and FDR < 0.05.

Gene expression RNAseq data of the training set were imported into the GSEA (version 4.3.0) in the JAVA environment to further investigate potential mechanisms and signaling pathways by which the two risk score subgroups participated. We classified the 242 patients into low- and high-risk score subgroups according to the median risk score. During execution process, “c2.cp.kegg.v7.0.symbols.gmt” is defined as the significant set. The enriched signaling items with adjusted p less than 0.05 and FDR q less than 0.25 were selected.

### Estimation of immune microenvironment

The estimation of the immune microenvironment was conducted by analyzing transcriptomic data from the TCGA cohort with 374 HCC patients. CIBERSORT software was employed to count the abundance of individual types of immunocytes in the whole cell population in each HCC sample. [62]. Next, we conducted the ssGSEA using the “GSVA” package for clarification of immunocytes infiltration in each sample. We utilized a two-tailed Spearman test to elucidate links between risk score and the fraction of 22 infiltrating immunocytes.

### Statistical analysis

R (version 4.2.3) with corresponding packages were used to conduct statistical analysis and draw statistical pictures. Two-tailed Chi-square testing or Fisher’s exact method were utilized to compare the clinicopathological outcome in two risk score subgroups. The survival probabilities of two subgroup patients were evaluated by employing K-M curves with log-rank test. The independent risk factors of the worse prognosis were recognized by the Multivariate Harzad-Cox model. The time-dependent ROC with AUC was used for the evaluation of the prediction capacity of the prognostic SRGs signature.

### Data availability statement

Transcription profiling data and corresponding clinical data of GSE14520 and GSE76427, and LIHC liver cancer dataset can obtain from the GEO database (GSE14520: (https://ftp.ncbi.nlm.nih.gov/geo/series/GSE14nnn/GSE14520/matrix/), GSE76427: (https://ftp.ncbi.nlm.nih.gov/geo/series/GSE76nnn/GSE76427/matrix/)) and the TCGA database (https://xenabrowser.net/datapages/?dataset=TCGA-LIHC.htseq_counts.tsv&host=https%3A%2F%2Fgdc.xenahubs.net&removeHub=https%3A%2F%2Fxena.treehouse.gi.ucsc.edu%3A443).
